# Pharmacovigilance practice on off-label high-dose of vancomycin in severely ill children

**DOI:** 10.3389/fphar.2025.1680239

**Published:** 2025-09-10

**Authors:** Min Qin, Wenwen Chen, Bin Lu, Yi Deng

**Affiliations:** ^1^ Department of Pharmacy, Chengdu Women’s and Children’s Central Hospital, School of Medicine, University of Electronic Science and Technology of China, Chengdu, China; ^2^ Pediatric Intensive Care Unit, Chengdu Women’s and Children’s Central Hospital, School of Medicine, University of Electronic Science and Technology of China, Chengdu, China

**Keywords:** vancomycin, off-label, acute kidney injury, child, pharmacovigilance

## Abstract

**Purpose:**

To investigate the safety of off-label high-dose vancomycin use in severely ill children and explore strategies to control risks.

**Methods:**

A case of acute kidney injury caused by off-label high-dose vancomycin (exceeding 40 mg/kg/day) in the Pediatric Intensive Care Unit (PICU) was analyzed. A retrospective case-control review of vancomycin treatment in 39 PICU patients from January to June 2020 were conducted.

**Findings:**

Among the 39 patients, 20 (51.3%) received off-label high-dose vancomycin dosing, and only 53.8% (21/39) underwent blood concentration testing. Five patients (25%; 5/20) in the off-label use group experienced vancomycin-associated adverse reactions, including three cases of severe ones. Based on risk factor analysis, we implemented improvement measures such as increasing the monitoring of vancomycin trough serum concentration, providing individualized drug regimens, enhancing training, and formally documenting off-label drug use. After implementing these measures from July 2020 to December 2022, a total of 86 children in the PICU were treated with vancomycin, and no cases of vancomycin-associated acute kidney injury was observed. The monitoring rate of vancomycin blood concentration increased to 88.4% (76/86).

**Implications:**

There is a risk of renal function damage when using high dosage of vancomycin in severely ill children, and healthcare providers should pay special attention to this in clinical medication safety practices.

## 1 Introduction

Vancomycin, a glycopeptide antibiotic, exerts its antibacterial effect by inhibiting the synthesis of bacterial cell walls and is a crucial drug for treating severe Gram-positive bacterial infections (Scholar, 2007). Due to the increasing number of nosocomial infections caused by drug-resistant *Staphylococcus aureus* and coagulase-negative staphylococci, vancomycin has been widely used in infants and children (Downes et al., 2017; Diorio et al., 2018; Jernigan et al., 2020) Although consensus has been reached on the dosing regimen and therapeutic drug range of vancomycin for adult patients, there is still debate regarding the ideal dosing regimen and optimal blood drug concentration of vancomycin for premature infants, term newborns, critically ill children, and obese children (Sosnin et al., 2019; Sridharan et al., 2019; Issaranggoon Na Ayuthaya et al., 2020; Oskarsdottir et al., 2021; Smit et al., 2021). Simultaneously, the impact of vancomycin on renal function in children is increasingly garnering attention. It has been reported that intravenous administration of vancomycin and piperacillin/tazobactam to critically ill children may increase the risk of acute kidney injury (Downes et al., 2017), and children with renal insufficiency are prone to acute kidney injury caused by vancomycin (Zhang et al., 2021). Therefore, it is necessary to monitor vancomycin concentrations in pediatric patients, especially in critically ill children.

An office of children and women’s Pharmacovigilance was set up by Chengdu Adverse Reaction Center and Chengdu Women’s and Children’s Central Hospital in 2019. The office consists of 1 medication safety officer (MSO) and 4 vigilance pharmacists, and they are responsible for the pharmacovigilance work of the whole hospital. A system has been established to identify signals of adverse drug events from abnormal drug concentration monitoring results and implement clinical medication interventions in our hospital. Through continuous exploration and improvement, the hospital has reduced the incidence of adverse drug events and formed a relatively comprehensive medication safety monitoring system for special populations, effectively improving the safe medication environment within the hospital. An event of vancomycin-associated acute kidney injure was noticed by the office staff. In response, a systematic investigation and targeted intervention were conducted to address risk factors associated with vancomycin administration in critically ill children at our institution.

## 2 Participants and methods

### 2.1 Review an acute kidney injury case caused by off-label high-dose vancomycin

A 6.2-kg infant with a history of live-donor liver transplantation for congenital biliary atresia, maintained on immunosuppression, was admitted to the PICU with severe pneumonia and acute respiratory failure. The patient exhibited 1 week of tachypnea, occasional cough, recurrent fever (peaking at 39.4 °C), and a transient maculopapular rash. Symptoms deteriorated 1 day prior to admission with increased work of breathing and reduced oral intake. The rash resolved following loratadine administration, while fever responded to ibuprofen. The child had no known drug allergies. Past medical history included neonatal jaundice and liver transplantation; immunosuppressants were continued until admission without specification. Key admission diagnoses comprised severe pneumonia, respiratory failure, moderate-to-severe dehydration, and moderate anemia. No adverse drug reactions (ADRs) were suspected during this presentation.

Upon admission, the infant’s vital signs included a temperature of 38.4 °C, heart rate of 201 bpm, respiratory rate of 52 breaths/min, and blood pressure of 111/69 mmHg. The patient appeared irritable with poor consolability, mild perioral cyanosis, poor skin turgor, and diminished tearing and salivation. Coarse breath sounds accompanied by medium to coarse rales were noted on lung auscultation. An abdominal surgical scar was also observed. Initial serum creatinine levels fell within the normal range.

The patient was treated with meropenem (120 mg IV q8h), budesonide (1 mg nebulized q6h), and acetylcysteine (150 mg nebulized q8h), in addition to supportive medications. Voriconazole (25 mg PO q12h) was introduced on hospital day 3. Vancomycin (90 mg IV q6h) was added on day 5, at which point baseline serum creatinine measured 45.4 μmol/L.

Two days after initiating vancomycin, the trough serum concentration level was elevated at 21.8 μg/mL (therapeutic range: 5–10 μg/mL (Clinical Pharmacology Group of Pediatric Branch of the Chinese Medical Association, 2015). Serum creatinine concurrently increased to 65.1 μmol/L (reference range: 17.3–54.6 μmol/L), consistent with acute kidney injury. Vancomycin was discontinued and replaced with linezolid; creatinine normalized to 26.6 μmol/L within 2 days, supporting a diagnosis of vancomycin-associated nephrotoxicity. A pharmacovigilance review was initiated to assess drug-induced renal impairment.

### 2.2 Investigation of risk factors of vancomycin used in severely ill children

Our hospital’s PICU is responsible for the transfer of critically ill children within the province and the city, as well as emergency rescue efforts for severe cases in the hospital. The most common adverse reaction to vancomycin in our hospital is various types of rashes, followed by red man syndrome, liver and kidney function damage, and fever. Although the number of cases of liver and kidney function damage is small, it has a significant impact on the growth and development of children, so attention should be paid to the safety of medication for children in clinical practice.

**FIGURE 1:**
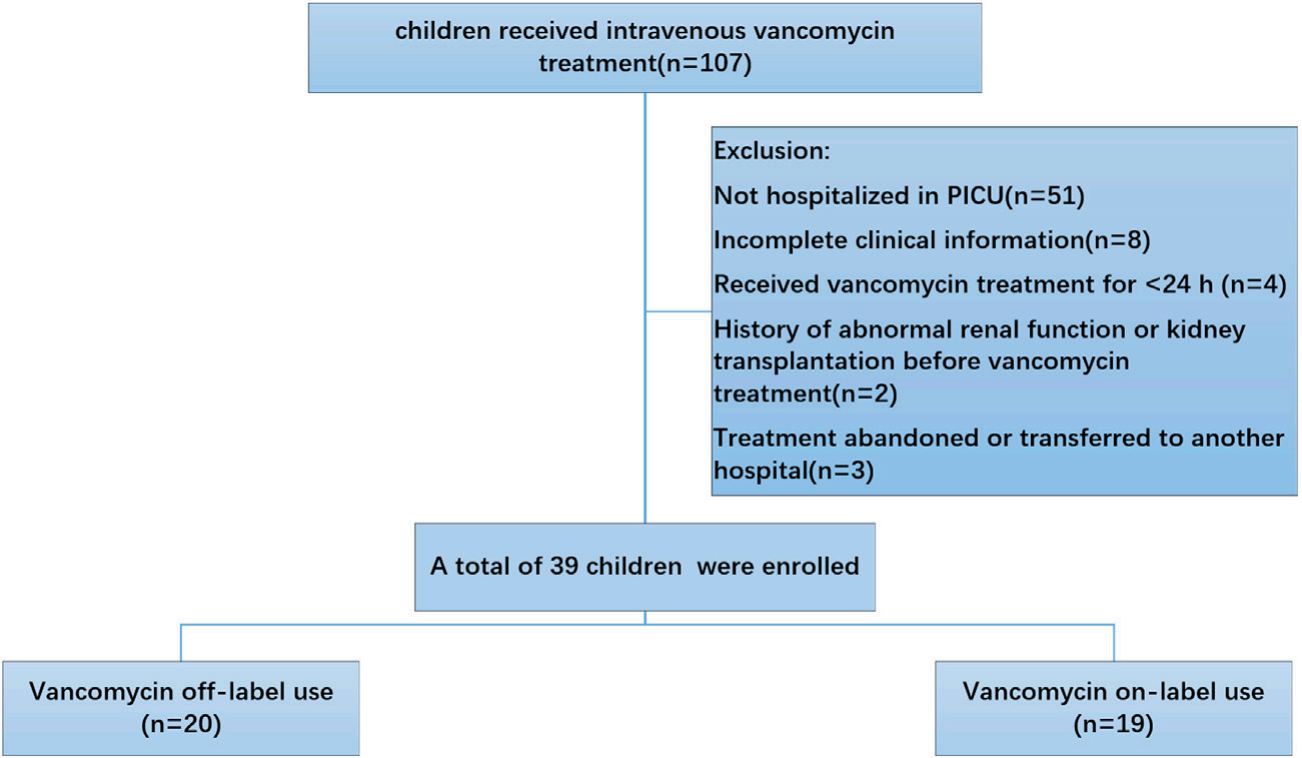
The schematic diagram of included patients.

To understand the vancomycin-associated adverse reactions in our hospital, we investigated the number of children who were administrated vancomycin. The schematic diagram of included patients was showed in Figure 1.

#### 2.2.1 Patients and methods

Inclusion criteria: (1) Inpatient in our hospital from January 2020 to June 2020; (2) Received vancomycin treatment for ≥24 h; (3) Aged between 0 and 18 years, with no gender restriction.

Exclusion criteria: (1) Cases with incomplete clinical data (e.g., no clear records of weight, premature birth status, etc.); (2) History of abnormal renal function or kidney transplantation before vancomycin treatment; (3) Not hospitalized in PICU.

Included patients were divided into two groups according to the dosage of vancomycin. The dosage of vancomycin off-label use group was above 40 mg/kg/day, while on-label use group was below 40 mg/kg/day. The efficacy of each drug was assessed based on whether the expected effect of the drug was observed. To assess the safety, we evaluated not only the reported ADRs but also events that occurred during the administration of vancomycin. According to KDIGO Criteria, vancomycin-associated acute kidney injury were defined an increase in serum creatinine by ≥ 0.3 mg/dL (≥26.5 μmol/L) within 48 h, or an increase in serum creatinine to ≥1.5 times baseline.

We obtained a list of patients’ data including patient demographics, diagnoses, reasons for administration, weight, dosage and duration of vancomycin treatment, vancomycin trough serum concentration and details of ADRs. The age of the patients was categorized as follows: 0–1 year, 1–3 years, 4–7 years, 8–12 years and 12–18 years. The primary outcomes were the efficacy and safety of the drugs, including mortality.

#### 2.2.2 Statistic methods

Descriptive analyses were performed to analyze the continuous variables. Categorical variables are expressed as numbers and percentages, and continuous variables as mean (standard deviation [SD]) or medians with the interquartile range. Student’s t-test or Mann-Whitney rank-sum test was used to test the statistical significance of continuous data. Binary logistic regression analysis was performed to evaluate the association between parameters and adverse drug events. All analyses were performed using Excel 2019 for Windows and SPSS ver. 22 (IBM Corp., Armonk, NY, United States of America). A threshold of P < 0.05 was set to indicate statistical significance.

## 3 Results

### 3.1 Basic information of included patients

Based on the above inclusion and exclusion criteria, 39 pediatric patients were finally included in the analysis. Basic information, underlying diseases, vancomycin usage (dose, duration of treatment), detection results of vancomycin trough serum concentration, vancomycin-associated adverse reactions, and treatment outcomes were recorded. According to the vancomycin prescribing information, dose for pediatric and infant patients is 40 mg/kg per day, whereas newborns should receive 10–15 mg/kg per dose. In this survey, 6 patients (15.4%) received a lower dose than recommended, 13 patients (33.3%) received the recommended dose, and 20 patients (51.3%) received a higher dose than recommended. The maximum daily dose was 84.21 mg/kg, the minimum daily dose was 35.09 mg/kg, and the average dose was 46.30 ± 10.84 mg/kg. Table 1 shows the demographic and clinical characteristics of the included patients. The total number of cases was 39. Gender factor had no clinically significant difference between off-label use and on-label use of vancomycin. Some parameters in the studied table showed a significant difference when comparing off-label use with on-label use of vancomycin; these were steady-state dosage, mean age, weight, outcome and adverse reactions. Other parameters showed a non-significant difference when comparing off-label use with on-label use of vancomycin; these were gender, duration of vancomycin therapy and Infection types.

**TABLE 1:** Demographic and clinical characteristics of the patients.

Group		Vancomycin off-label use (>40 mg/kg/day)	Vancomycin on-label use (≤40 mg/kg/day)	P
Sample size		20	19	
Steady-state dosage (mg/kg/d)		38.9 ± 1.8	53.3 ± 11.2	0.000^a^
Age (years)		1.8 ± 2.8	5.2 ± 4.5	0.007^a^
	<1, *n* (%)	13 (65.0%)	6 (31.6%)	
	1–3, *n* (%)	4 (20.0%)	4 (21.1%)	
	4–7, *n* (%)	2 (10.0%)	2 (10.5%)	
	8–12, *n* (%)	0 (0.0%)	2 (10.5%)	
	>12, *n* (%)	1 (5.0%)	5 (26.3%)	
Gender				
	Male, *n* (%)	13 (65%)	9 (47.4%)	0.267
	Female, *n* (%)	7 (35%)	10 (25.6%)	
Duration of vancomycin therapy (days)		9.1 ± 6.5	11.3 ± 8.3	0.267
Weight (kg), IQR		6.65 (3.3, 23.5)	15 (3,57)	0.010^a^
Outcome				
	Good outcome^a^, *n* (%)	14 (70%)	18 (94.7%)	0.000^a^
	Poor outcome^a^, *n* (%)	6 (30%)	1 (5.3%)	
Adverse reactions				
	Nephrotoxicity, *n* (%)	1 (5.0%)	0	0.047^a^
	Hepatotoxicity, *n* (%)	1 (5.0%)	0	
	Red man syndrome, *n* (%)	1 (5.0%)	0	
	Drug fever, *n* (%)	1 (5.0%)	0	
	Rash, *n *(%)	1 (5.0%)	0	
Infection type, *n* (%)				
	Septicopyemia, *n* (%)	9 (45.0%)	7 (36.8%)	0.605
	Pneumonia, *n* (%)	13 (65.0%)	11 (57.9%)	0.648
	Meningitis, *n* (%)	7 (35.0%)	2 (10.5%)	0.070
	Dermal infection, *n* (%)	2 (10.0%)	2 (10.5%)	0.957
	Urinary tract infection, *n* (%)	1 (5.0%)	1 (5.3%)	0.970
	Endocarditis, *n* (%)	1 (5.0%)	0 (0.0%)	0.323

^a^ Good outcome: recovery or improvement; Poor outcome: AKI, or lack of improvement.

### 3.2 Vancomycin-associated adverse reactions in our hospital

39 pediatric patients in the PICU received vancomycin treatment, accounting for 43.32% of the total vancomycin usage in the hospital. Five cases of vancomycin-associated adverse reactions were reported, including three severe adverse reactions: one case each of liver function damage, kidney function damage, and red man syndrome. These accounted for 83.3% of the vancomycin-associated adverse reactions reported in the hospital in 2020. The basic information of the cases with severe adverse reactions is shown in Table 2.

**TABLE 2:** Basic Information of Cases with severe Adverse Reactions.

ADRs	Age (days)	Weight (kg)	Single dose of vancomincy (mg)	Frequency	dosage (mg/kg/day)
Red man syndrome	49	4.36	50	q6h	45.87
Liver function lesion	46	3.6	50	q6h	55.56
vancomycin-associated nephrotoxicity	246	6.2	90	q6h	58.08

### 3.3 Monitoring of vancomycin concentration in PICU

**FIGURE 2:**
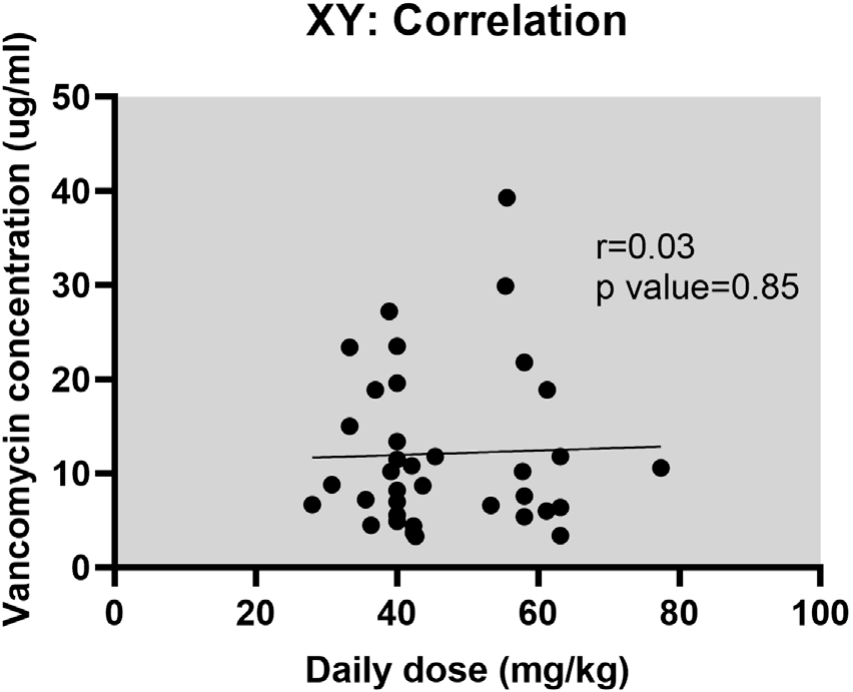
The correlation between vancomycin dosage and concentration (r = 0.03, p = 0.85).

In this study, the duration of vancomycin treatment ranged from 2 to 36 days, with a median duration of 8 days. Thirty patients (76.9%) received treatment for more than 3 days. According to the “Expert Consensus on Therapeutic Drug Monitoring for Children” (2015) (11), blood concentration monitoring is recommended for children who have been on vancomycin for more than 3 days, have severe infections, unstable renal function, or are concomitantly using other nephrotoxic or ototoxic drugs. Actual therapeutic drug monitoring of vancomycin was performed on 21 cases (41 times), with a blood drug concentration submission rate of 53.8% (21/39). Adjustments of vancomycin dosage were made in 14 cases after monitoring. Table 3 presents the details of the 21 patients who underwent vancomycin concentration monitoring. The correlation between monitoring results and vancomycin dosage is illustrated in Figure 2, which shows that the vancomycin concentrations *in vivo* do not correlate with the dose administered.

**TABLE 3:** Basic information of patients monitored for vancomycin drug concentration [*n* (%)].

	Max Value	Min Value	Average Value
Age (days)	4,267	63	1179.46 ± 1513.42
Duration of medication (days)	35	2	14.76 ± 6.56
Daily dose (mg/kg)	77.42	28	46.57 ± 11.63
Vancomycin concentration (ug/mL)	39.3	3.3	14.04 ± 9.61

### 3.4 Risk factor analysis of vancomycin therapy in severely ill children

#### 3.4.1 Risks associated with characteristic of vancomycin

Vancomycin belongs to the category of high-alert medications for children in China, with the primary risk points being nephrotoxicity and ototoxicity. The recommended strategy involves its application under the guidance of infectious disease specialists, strictly adhering to its indications, and closely monitoring blood drug concentrations (Wang et al., 2017). In the early stages of vancomycin development, its severe toxicity was associated with contaminants in the drug preparation. However, with continuous improvements in preparation techniques, the toxicity related to drug impurities has been significantly reduced (Darko and Guharoy, 2003). Reports indicate that a vancomycin concentration of 10 mg/L is associated with increased risks of treatment failure and antibiotic resistance, while concentrations exceeding 20 mg/L may increase the likelihood of nephrotoxicity (Liu et al., 2011; Elyasi et al., 2012; Jung et al., 2014). This is particularly relevant in critically ill patients with dynamic pharmacokinetics, where trough serum concentrations may not reliably reflect AUC targets. Bayesian approaches enable AUC estimation with limited blood samples, facilitating individualized dosing in children where frequent sampling is challenging. Therefore, it is crucial to revisit the toxicity of vancomycin in the clinical setting, especially for severely ill children.

#### 3.4.2 Physiopathological risks in severely ill children

The pediatric population varies widely in age, body size, and developmental stage, which also affects changes in pharmacokinetics and pharmacodynamics (Thakkar et al., 2017). Due to variations in renal clearance and distribution volume, it is more difficult for children to achieve and maintain the desired serum concentrations of vancomycin compared to adults, and this challenge is often exacerbated in severely ill pediatric patients. Changes such as capillary leakage, fluid shifts, alterations in renal clearance (both increases and decreases), variations in protein concentration and drug binding, and modifications in liver function can lead to significant variations in vancomycin serum concentrations in severely ill children, even at standard doses (Kloprogge et al., 2019). Therapeutic interventions like renal replacement therapy, extracorporeal membrane oxygenation, administration of blood products, use of vasoactive agents, and administration of multiple drugs may all result in notable pharmacodynamic changes. The existing standard dosing guidelines for vancomycin in children have not been specifically validated in critically ill patients. These patients likely exhibit different drug distribution and elimination patterns compared to the general population, affecting the calculation of crucial pharmacokinetic parameters such as AUC, which warrants further investigation (Mahmood, 2014). Consequently, achieving optimal therapeutic vancomycin levels may rely on continuous monitoring, corresponding dose adjustments, and clinical response to treatment.

#### 3.4.3 Risks of off-label dosing

In this investigation, it was observed that off-label dosing of vancomycin is a relatively common practice in the clinical management of severely ill children, posing risks for both physicians and patients. The new “Medical Practitioners Law” stipulates that off-label drug use can only be initiated “under special circumstances where there are no effective or better treatment options available,” and it should be accompanied by strict informed consent, monitoring of adverse reactions, and proactive treatment. The 2020 consensus guidelines for vancomycin therapeutic drug monitoring, updated by the American Society of Health-System Pharmacists (ASHP), the Infectious Diseases Society of America (IDSA), the Pediatric Infectious Diseases Society (PIDS), and the Society of Infectious Diseases Pharmacists (SIDP) (Rybak et al., 2020), also mention that “the safety of vancomycin doses exceeding 80 mg˙kg-1˙d-1 in children has not been prospectively evaluated. It is advisable to avoid vancomycin doses ≥100 mg˙kg-1˙d-1, as they may exceed exposure thresholds.” The three cases of severe adverse reactions reported in the PICU all involved doses exceeding the recommended label, indicating that extra caution should be exercised when using off-label doses of vancomycin for anti-infective treatment in severely ill children. Monitoring serum creatinine concentrations and vancomycin trough serum concentration is recommended to ensure safety and efficacy.

## 4 Discussion

Based on the analysis of risk factors associated with vancomycin therapy, the following improvement measures are proposed: (1) Increase the monitoring rate of vancomycin blood concentration. PICU is required to monitor the trough serum concentration at least once during the course of treatment for children receiving vancomycin; (2) Pharmacists should combine monitoring results with the Bayesian method to calculate individual pharmacokinetic parameters for dose adjustment, providing individualized medication regimens; (3) Provide training for PICU physicians and nurses, covering vancomycin pharmacokinetics, pharmacodynamics, dosage and usage for children, medication precautions, effects of concomitant medications, selection of compatible solvents, preparation concentration, dripping speed, the correlation between drug concentration monitoring results and dosage, discussion of severe adverse reactions, and risks of off-label drug use; (4) Implement an off-label drug use record system for vancomycin overdose in severely ill children. If a patient indeed requires an off-label dose, detailed disclosure obligations must be fulfilled, explicit informed consent from the patient or their family members must be obtained, and a consent form must be signed. To assess the effectiveness of the interventions, we investigated the use of vancomycin among hospitalized children in PICU from July 2020 to December 2022 and compared it with data from January to June 2021 before the intervention. The results showed that 86 children received vancomycin treatment, and none of them experienced renal function damage related to off-label vancomycin dosing. The monitoring rate of vancomycin blood concentration increased from 53.8% (21/39) before the intervention to 88.4% (76/86) after the intervention, with a statistically significant difference (χ^2^ = 18.401, P < 0.01).

Based on a comprehensive review of the patient’s pharmacotherapy, the acute kidney injury was attributed to supratherapeutic vancomycin exposure resulting from off-label dosing. Our findings align with previous studies in children, which report substantial interindividual variability in vancomycin clearance—especially among critically ill patients—highlighting the inadequacy of trough-only monitoring (Akunne et al., 2022). This study is limited by its single-center design, small sample size, and dependence on trough serum concentrations rather than AUC-guided dosing, potentially affecting its generalizability and exposure accuracy. Future multi-center prospective trials should employ model-informed Bayesian estimation for AUC targeting to validate safer dosing strategies in this vulnerable population.

## 5 Conclusion

Vancomycin is widely used in the treatment of severely ill children, with clear clinical efficacy against methicillin-resistant *Staphylococcus aureus*, penicillin-resistant *Streptococcus* pneumoniae, and hospital-acquired infections. However, its nephrotoxicity and ototoxicity can have irreversible effects on children, especially in severely ill children where dose variations are significant. Additionally, off-label dosing practices exist in clinical settings, which can easily lead to adverse reactions if not managed properly. Therefore, pharmacists should pay special attention to the safe clinical use of vancomycin.

## Data Availability

The raw data supporting the conclusions of this article will be made available by the authors upon reasonable request.

## References

[B1] Akunne O. O. Mugabo P. Argent A. C. (2022). Pharmacokinetics of vancomycin in critically ill children: a systematic review. Eur. J. Drug Metab. Pharmacokinet. 47, 31–48. 10.1007/s13318-021-00730-z 34750740 PMC8574943

[B2] Clinical Pharmacology Group of Pediatric Branch of the Chinese Medical Association Subspecialty Group of Pediatric Clinical Pharmacology Society of Pediatrics Chinese Medical Association (2015). Expert consensus on therapeutic drug monitoring for children. Chin. J. Pediatr. 53, 650–659. 10.3760/cma.j.issn.0578-1310.2015.09.005

[B3] Darko W. Guharoy R. (2003). Mississippi mud no more: cost effectiveness of pharmacokinetic dosage adjustment of vancomycin to prevent nephrotoxicity. Pharmacotherapy 23, 8. 10.1592/phco.23.5.643.32199 12741439

[B4] Diorio C. Robinson P. D. Ammann R. A. Castagnola E. Erickson K. Esbenshade A. (2018). Guideline for the management of Clostridium difficile infection in children and adolescents with cancer and pediatric hematopoietic stem-cell transplantation recipients. J. Clin. Oncol. 36, 3162–3171. 10.1200/JCO.18.00407 30216124 PMC6209092

[B5] Downes K. J. Cowden C. Laskin B. L. Huang Y.-S. Gong W. Bryan M. (2017). Association of acute kidney injury with concomitant vancomycin and piperacillin/tazobactam treatment among hospitalized children. JAMA Pediatr. 171, e173219. 10.1001/jamapediatrics.2017.3219 28973124 PMC6583633

[B6] Elyasi S. Khalili H. Dashti-Khavidaki S. Mohammadpour A. (2012). Vancomycin-induced nephrotoxicity: mechanism, incidence, risk factors and special populations. A literature review. Eur. J. Clin. Pharmacol. 68, 1243–1255. 10.1007/s00228-012-1259-9 22411630

[B7] Issaranggoon Na Ayuthaya S. Katip W. Oberdorfer P. Lucksiri A. (2020). Correlation of the vancomycin 24-h area under the concentration-time curve (AUC24) and trough serum concentration in children with severe infection: a clinical pharmacokinetic study. Int. J. Infect. Dis. 92, 151–159. 10.1016/j.ijid.2019.12.036 31935538

[B8] Jernigan J. A. Hatfield K. M. Wolford H. Nelson R. E. Olubajo B. Reddy S. C. (2020). Multidrug-resistant bacterial infections in U.S. Hospitalized patients, 2012-2017. N. Engl. J. Med. 382, 1309–1319. 10.1056/NEJMoa1914433 32242356 PMC10961699

[B9] Jung Y. Song K.-H. Cho J. Kim H. Kim N.-H. Kim T. S. (2014). Area under the concentration-time curve to minimum inhibitory concentration ratio as a predictor of vancomycin treatment outcome in methicillin-resistant *Staphylococcus aureus* bacteraemia. Int. J. Antimicrob. Agents 43, 179–183. 10.1016/j.ijantimicag.2013.10.017 24315788

[B10] Kloprogge F. Hill L. F. Booth J. Klein N. Irwin A. D. Dixon G. (2019). Revising pediatric vancomycin dosing accounting for nephrotoxicity in a pharmacokinetic-pharmacodynamic model. Antimicrob. Agents Chemother. 63, e00067-19–19. 10.1128/AAC.00067-19 30833429 PMC6496060

[B11] Liu C. Bayer A. Cosgrove S. E. Daum R. S. Fridkin S. K. Gorwitz R. J. (2011). Clinical practice guidelines by the infectious diseases society of America for the treatment of methicillin-resistant *Staphylococcus aureus* infections in adults and children. Clin. Infect. Dis. 52, e18–e55. 10.1093/cid/ciq146 21208910

[B12] Mahmood I. (2014). Dosing in children: a critical review of the pharmacokinetic allometric scaling and modelling approaches in paediatric drug development and clinical settings. Clin. Pharmacokinet. 53, 327–346. 10.1007/s40262-014-0134-5 24515100

[B13] Oskarsdottir K. Haraldsson A. Thorkelsson T. Oskarsdottir T. Gunnarsson P. Thors V. (2021). Children may need higher vancomycin doses to achieve therapeutic levels. Acta Paediatr. 110, 3077–3082. 10.1111/apa.16025 34233034

[B14] Rybak M. J. Le J. Lodise T. P. Levine D. P. Bradley J. S. Liu C. (2020). Therapeutic monitoring of vancomycin for serious methicillin-resistant staphylococcus aureus infections: a revised consensus guideline and review by the American society of health-system pharmacists, the infectious diseases society of America, the pediatric infectious diseases society, and the society of infectious diseases pharmacists. Am. J. Health Syst. Pharm. 77, 835–864. 10.1093/ajhp/zxaa036 32191793

[B15] Scholar E. (2007). “Vancomycin,” in xPharm: the comprehensive Pharmacology reference. Editors Enna S. J. Bylund D. B. (New York: Elsevier), 1–6. 10.1016/B978-008055232-3.62843-0

[B16] Smit C. Goulooze S. C. Brüggemann R. J. M. Sherwin C. M. Knibbe C. A. J. (2021). Dosing recommendations for vancomycin in children and adolescents with varying levels of obesity and renal dysfunction: a population pharmacokinetic study in 1892 children aged 1-18 years. AAPS J. 23, 53. 10.1208/s12248-021-00577-x 33839974 PMC8038958

[B17] Sosnin N. Curtis N. Cranswick N. Chiletti R. Gwee A. (2019). Vancomycin is commonly under-dosed in critically ill children and neonates. Br. J. Clin. Pharmacol. 85, 2591–2598. 10.1111/bcp.14084 31378957 PMC6848905

[B18] Sridharan K. Al Daylami A. Ajjawi R. Al-Ajooz H. Veeramuthu S. (2019). Clinical pharmacokinetics of vancomycin in critically ill children. Eur. J. Drug Metab. Pharmacokinet. 44, 807–816. 10.1007/s13318-019-00568-6 31301023

[B19] Thakkar N. Salerno S. Hornik C. P. Gonzalez D. (2017). Clinical Pharmacology studies in critically ill children. Pharm. Res. 34, 7–24. 10.1007/s11095-016-2033-y 27585904 PMC5177463

[B20] Wang C. Wang X. Li X. Liang H. Wang Y. (2017). Initial development of a high-alert medication list for children in China. Adverse Drug React. J. 19, 10–16. 10.3760/cma.j.issn.1008-5734.2017.01.004

[B21] Zhang H. Gao P. Wang Y. Chen J. Jia G. Zhang F. (2021). Baseline kidney function is associated with vancomycin-induced acute kidney injury in children: a prospective nested case-control study. Pediatr. Nephrol. 36, 1299–1306. 10.1007/s00467-020-04820-z 33161460

